# PATTERNS AND ASSOCIATED FACTORS OF TECHNOLOGY USE IN US COMMUNITY-DWELLING OLDER ADULTS

**DOI:** 10.1093/geroni/igae098.2564

**Published:** 2024-12-31

**Authors:** Wenting Peng, Yuqian Luo, Minhui Liu

**Affiliations:** University of Washington, Seattle, Washington, United States; Hong Kong Polytechnic University, Hong Kong, Hong Kong; Ningxia Medical University, Yinchuan, Ningxia, China

## Abstract

Technology, especially digital health technology, has been increasingly used to deliver healthcare services. The COVID-19 pandemic accelerated the rapid adoption of medical technology. However, how many and how older people currently use technology remain unknown. Using the cross-sectional data from the National Health and Aging Trends Study collected in 2022, we examined the prevalence, characteristics, and associated factors of technology use (including technology device use, everyday/digital health technology use) among 5,512 community Medicare beneficiaries aged 65 years and older with analytical sample weights and design-based logistic regression models. Results showed that over 1.3 million (2.7%) older people did not use any information and communication technology devices (i.e., computers, cell phones, and tablets). The weighted prevalence of everyday technology use and digital health technology use were 83.3% (95% CI, 82.1%-84.5%) and 52.1% (95% CI, 49.5%-54.7%), respectively. Compared to individuals who never used digital health technology, older adults who used digital health technology were more likely to be younger, white, not living alone, self-rated as having good health, non-homebound, and possessed higher education, income, and cognitive function, with fewer ADL/IADL impairments, and were less likely to experience hospitalization and depressive symptoms (All P< 0.05). Age, education, income and a diagnosis of dementia were independently associated with technology device use, everyday technology use, and digital health technology use (All P< 0.05). The prevalence of technology use in US older adults differed significantly by demographic and health-related characteristics. Future technology-based services or interventional studies should consider the devices and modalities of technologies in the older population.

